# Correlative intravital imaging of cGMP signals and vasodilation in mice

**DOI:** 10.3389/fphys.2014.00394

**Published:** 2014-10-14

**Authors:** Martin Thunemann, Kjestine Schmidt, Cor de Wit, Xiaoxing Han, Rakesh K. Jain, Dai Fukumura, Robert Feil

**Affiliations:** ^1^Interfakultäres Institut für Biochemie, University of TübingenTübingen, Germany; ^2^Institut für Physiologie, Universität zu LübeckLübeck, Germany; ^3^Edwin L. Steele Laboratory, Department of Radiation Oncology, Massachusetts General Hospital and Harvard Medical SchoolBoston, MA, USA

**Keywords:** biosensor, cremaster, cyclic GMP, dorsal skinfold chamber, fluorescence resonance energy transfer, intravital imaging, microcirculation, multiphoton microscopy

## Abstract

Cyclic guanosine monophosphate (cGMP) is an important signaling molecule and drug target in the cardiovascular system. It is well known that stimulation of the vascular nitric oxide (NO)-cGMP pathway results in vasodilation. However, the spatiotemporal dynamics of cGMP signals themselves and the cGMP concentrations within specific cardiovascular cell types in health, disease, and during pharmacotherapy with cGMP-elevating drugs are largely unknown. To facilitate the analysis of cGMP signaling *in vivo*, we have generated transgenic mice that express fluorescence resonance energy transfer (FRET)-based cGMP sensor proteins. Here, we describe two models of intravital FRET/cGMP imaging in the vasculature of cGMP sensor mice: (1) epifluorescence-based ratio imaging in resistance-type vessels of the cremaster muscle and (2) ratio imaging by multiphoton microscopy within the walls of subcutaneous blood vessels accessed through a dorsal skinfold chamber. Both methods allow simultaneous monitoring of NO-induced cGMP transients and vasodilation in living mice. Detailed protocols of all steps necessary to perform and evaluate intravital imaging experiments of the vasculature of anesthetized mice including surgery, imaging, and data evaluation are provided. An image segmentation approach is described to estimate FRET/cGMP changes within moving structures such as the vessel wall during vasodilation. The methods presented herein should be useful to visualize cGMP or other biochemical signals that are detectable with FRET-based biosensors, such as cyclic adenosine monophosphate or Ca^2+^, and to correlate them with respective vascular responses. With further refinement and combination of transgenic mouse models and intravital imaging technologies, we envision an exciting future, in which we are able to “watch” biochemistry, (patho-)physiology, and pharmacotherapy in the context of a living mammalian organism.

## Introduction

Blood vessels are highly organized structures consisting of various cell types including endothelial cells in the intima, smooth muscle cells (SMCs) in the media, and fibroblasts in the adventitia. These cells receive signals from surrounding tissue, blood, and the nervous system, and they interact with each other in multiple ways. Using *ex vivo* or *in vitro* systems, it is challenging, if not impossible, to mimic the vessel's three-dimensional structure, microenvironment, and physiological control systems present *in vivo*. Current research aims to understand biochemical pathways contributing to vascular function and dysfunction in health and disease. Intravital microscopy (IVM) is a powerful tool to study signaling processes in vascular cells while still in their natural environment. IVM-based approaches have been developed to analyze vascular structure and hemodynamic parameters *in vivo*, mainly in surgically exposed tissues or through implanted optical windows (Weigert et al., 2013). Technical innovations in confocal and multiphoton (MP) microscopy and the generation of transgenic animals expressing fluorescent proteins paved the way for state-of-the-art IVM (Jain et al., 2002; Kirkpatrick et al., 2012). In combination with motion compensation, IVM can achieve spatiotemporal resolutions similar to microscopy under *in vitro* conditions (Lee et al., 2014).

Ca^2+^, cyclic adenosine monophosphate (cAMP), and cyclic guanosine monophosphate (cGMP) are key players in the regulatory network of vascular function (Carvajal et al., 2000; De Wit et al., 2000; Somlyo and Somlyo, 2000; Berridge, 2008; Morgado et al., 2012). Transgenic mice were generated that express fluorescent Ca^2+^ indicator proteins in endothelial cells or SMCs (Isotani et al., 2004; Tallini et al., 2007; Mauban et al., 2013) and cardiomyocytes (reviewed in Kaestner et al., 2014). Interestingly, Mauban et al. recently reported that spontaneous high-amplitude Ca^2+^ waves do not occur in SMCs of resistance-type vessels *in vivo*, although these waves have been observed before in isolated vessels of various origin (Mauban et al., 2013). This finding challenges the hypothesis that high-amplitude Ca^2+^ waves significantly contribute to the regulation of basal vascular tone *in vivo* (Mauban et al., 2013) and highlights the importance to study cardiovascular physiology *in vivo* (Wier, 2014). In contrast to Ca^2+^, vascular cAMP and cGMP signals have not been visualized *in vivo* until now, although respective biosensors have been developed and used in cells and isolated tissues (Zaccolo et al., 2005; Nikolaev and Lohse, 2009; Thunemann et al., 2013a). These experiments indicated the existence of subcellular cyclic nucleotide compartments in cardiomyocytes and vascular SMCs and a cross-modulation of cAMP and cGMP signals *in vitro* (Fischmeister et al., 2006; Zaccolo and Movsesian, 2007; Nausch et al., 2008; Stangherlin and Zaccolo, 2012). Localized signaling domains might play important roles in cardiovascular function and dysfunction (Nikolaev et al., 2010). *In vitro* experiments have also shown substantial crosstalk between Ca^2+^ and cyclic nucleotide signaling pathways (Carvajal et al., 2000; Feil et al., 2003). However, the relevance of these mechanisms for the regulation of vascular tone *in vivo* has yet to be demonstrated.

Cyclic GMP is generated by guanylyl cyclases in response to nitric oxide (NO) and natriuretic peptides. Its effects are mediated through cGMP-dependent protein kinases (cGKs), cyclic nucleotide-gated ion channels, and cyclic nucleotide-degrading phosphodiesterases. A key function of cGMP in the vasculature is the induction of vasodilation, perhaps by modulating Ca^2+^ signaling pathways in vascular SMCs (Lincoln et al., 2001; Beavo and Brunton, 2002; Hofmann et al., 2006; Kemp-Harper and Feil, 2008; Francis et al., 2011). Indeed, IVM of the cremaster microcirculation demonstrated the importance of the NO-cGMP-cGK pathway for dilation of resistance-type vessels *in vivo* (De Wit et al., 1994; Koeppen et al., 2004). Moreover, natriuretic peptide-induced cGMP modulates endothelial permeability, therefore contributing to the regulation of blood volume and pressure (Kuhn, 2012). Cyclic GMP also mediates the effects of drugs used to treat angina pectoris (e.g., organic nitrates), erectile dysfunction (e.g., sildenafil, tadalafil), and pulmonary hypertension (e.g., sildenafil, riociguat) (Kemp-Harper and Feil, 2008; Schlossmann and Schinner, 2012). Although considerable progress has been made in unraveling cGMP's roles in cardiovascular physiology and pathophysiology, many of its functions remain controversial (Feil et al., 2003, 2005; Tsai and Kass, 2009).

To extend our understanding of cardiovascular cGMP signaling *in vivo*, we sought to establish methods that allow the visualization of cGMP in living mice. Then, we would be able to correlate cGMP signals with functional responses, such as vasodilation, under physiological and pathophysiological conditions, or upon pharmacotherapy with cGMP-elevating drugs. Therefore, we generated transgenic mice expressing cGMP biosensors based on fluorescence (or Förster) resonance energy transfer (FRET) and reported for the first time on intravital FRET/cGMP imaging in living mice (Thunemann et al., 2013b).

## Methodology

This section describes the steps necessary to perform and evaluate IVM experiments in the vasculature of anesthetized mice expressing FRET-based cGMP biosensors. We also describe an evaluation routine that allows estimating FRET/cGMP changes in moving structures, such as dilating vessels. These methods are likely adaptable to experiments within other vascular beds, e.g., in mesenteric, femoral, or cerebral arteries, or with mice expressing other FRET-based biosensors.

### cGMP biosensor

**Figure 1:**
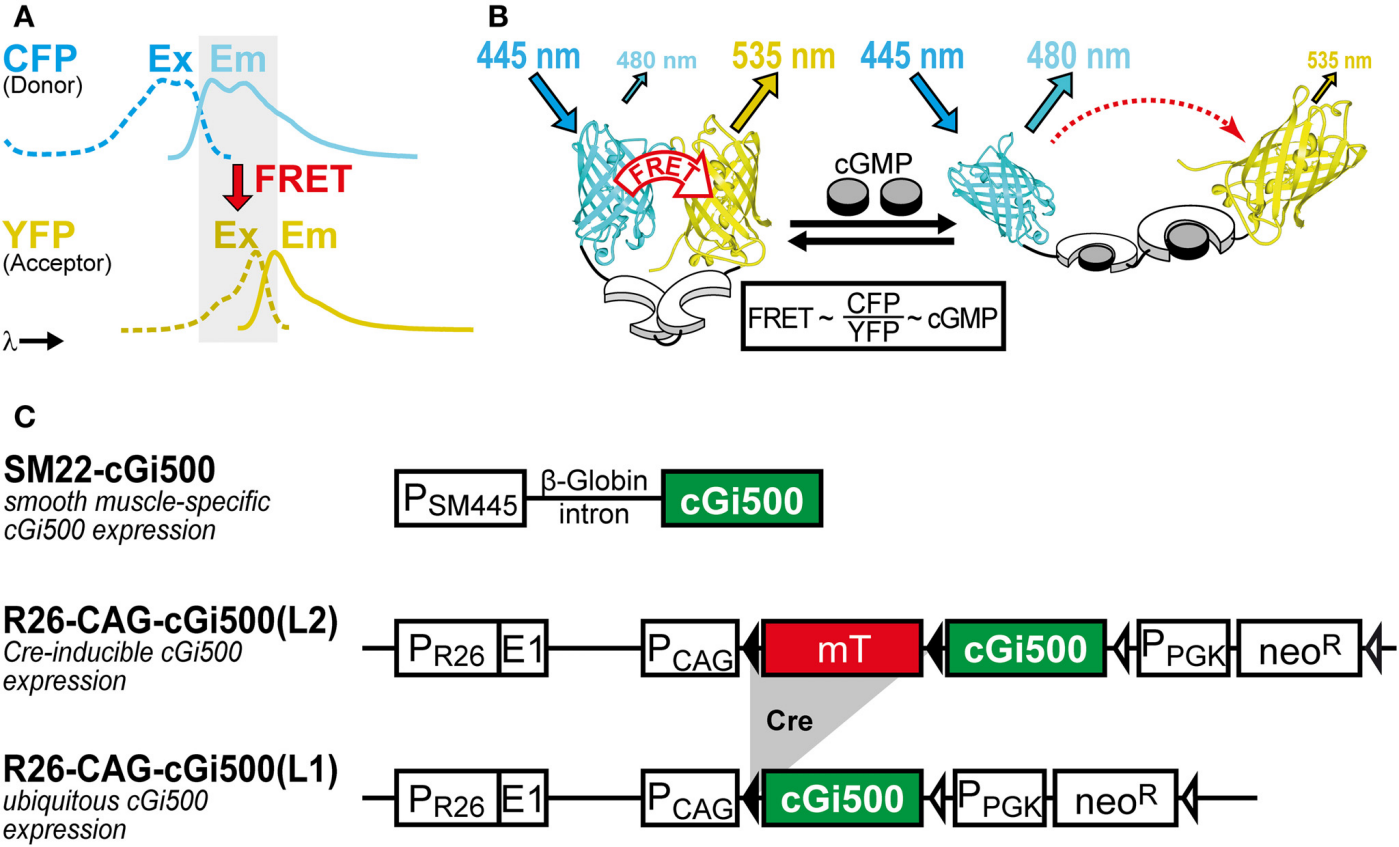
**(A,B)** Working principle of the FRET-based cGi500 biosensor and **(C)** transgenes used to generate cGi500-expressing mice. **(A)** Spectral overlap (gray) of YFP excitation (Ex, dashed lines) and CFP emission (Em, solid lines) spectra that is necessary for FRET to occur. **(B)** The cGMP indicator protein cGi500 consists of the tandem cGMP-binding sites from bovine cGK type I (white) flanked by CFP and YFP. Without cGMP, FRET occurs from excited CFP to YFP, leading to light emission from YFP. Binding of cGMP (gray) causes a conformational change and a decrease in FRET efficiency, so that light emission from YFP at 535 nm is reduced and emission from CFP at 480 nm is increased. **(B)** is reproduced from Thunemann et al. (2013b). **(C)** Constructs used to generate transgenic cGi500-expressing mice. Abbreviations: E1, first exon of the endogenous Rosa26 gene; mT, membrane-targeted tandem-dimer tomato red fluorescent protein; neo^R^, neomycin resistance gene; P_CAG_, chicken actin/β-globin promoter; P_PGK_, phosphoglycerate kinase promoter; P_SM445_, 445-bp promoter fragment of the Transgelin/SM22 gene; P_R26_, endogenous Rosa26 promoter. Black triangles represent loxP sites, open triangles represent FRT sites. See also Section “Selection of Appropriate Biosensors and Generation of Biosensor-Expressing Mice” for further details on mouse generation and characterization.

We use the FRET-based cGMP biosensor cGi500 (“cGMP indicator with an EC_50_ of 500 nM”) generated by Russwurm et al. (2007). This monomolecular biosensor consists of the two cGMP-binding sites from bovine cGK type I sandwiched between cyan and yellow fluorescent protein (CFP and YFP). Detection of cGMP with cGi500 is based on FRET from CFP to YFP, whose spectra show sufficient overlap for efficient FRET (Figure 1A). In the absence of cGMP, CFP and YFP are in favorable distance and orientation for FRET to occur, whereas cGMP binding induces a reversible structural change of cGi500 that reduces FRET efficiency (Figure 1B). The ratio of CFP and YFP fluorescence (R = CFP/YFP) recorded upon CFP excitation can be taken as a measure for the FRET efficiency (Clegg, 2009) and, therefore, for intracellular cGMP concentrations measured with cGi500. It has been shown in sensor-containing cell extracts and escin-permeabilized cells that cGi500 detects cGMP concentrations as low as 50–100 nM and is saturated at approx. 3 μM cGMP. Similar experiments have shown that cGi500 detects cAMP only at levels higher than 30 μM, which exceeds typical physiological cAMP concentrations (Russwurm et al., 2007; Thunemann et al., 2013a; Milde and Feil, unpublished results). Based on its high selectivity for cGMP over cAMP, its dynamic range for cGMP detection, fast kinetics, and relatively large CFP/YFP ratio changes of up to ~50%, the cGi500 sensor appears to be suitable for *in vivo* use.

### Transgenic mice

Animal experiments were performed in compliance with the guidelines for human care and use of laboratory animals. They were approved by the Regierungspräsidium Tübingen, the Ministerium für Energiewende, Landwirtschaft, Umwelt und ländliche Räume des Landes Schleswig-Holstein, and local authorities in Boston, MA. The generation of transgenic cGMP sensor mice has been described previously (Thunemann et al., 2013b). Transgene structures are shown in Figure 1C. Briefly, the SM22-cGi500 mouse line was generated by random transgenesis using a 445-bp promoter fragment of the Transgelin/SM22 gene (Li et al., 1996) to drive cGi500 expression. The R26-CAG-cGi500(L2) and R26-CAG-cGi500(L1) lines were generated by targeted mutagenesis of the Rosa26 (R26) locus with a construct derived from the R26-mT/mG targeting vector (Muzumdar et al., 2007). IVM data shown and discussed in this article were obtained with the R26-CAG-cGi500(L1) mouse line that expresses cGi500 ubiquitously (Thunemann et al., 2013b).

### Epifluorescence cGMP imaging in the cremaster microcirculation

#### Imaging setup

- Microscope: upright Axioplan II FS (Carl Zeiss Microscopy) with Achroplan 40×/0.75 N.A. water immersion lens. The system is placed on a motorized shifting table (Luigs and Neumann).
- Light source: Polychrome V (FEI Munich) set to 420 nm or Spectra X with 438/24 nm light engine (Lumencor).
- Filters: 470 nm dichroic mirror (AHF). Beam splitter: Micro-Imager DUAL-View with 516 nm dichroic mirror, 480/50 and 535/40 nm emission filters (Photometrics).
- Detector: Andor iXon 885 (Andor Technology) or Quant-EM 512sc (Photometrics) EM-CCD camera.
- Software: Live Acquisition (FEI Munich) or VisiView (Visitron Systems).

#### Surgery

All surgical procedures have to be approved by the Institutional Animal Care and Use Committee. Male mice are required for IVM of the cremaster microcirculation.

**Figure 2:**
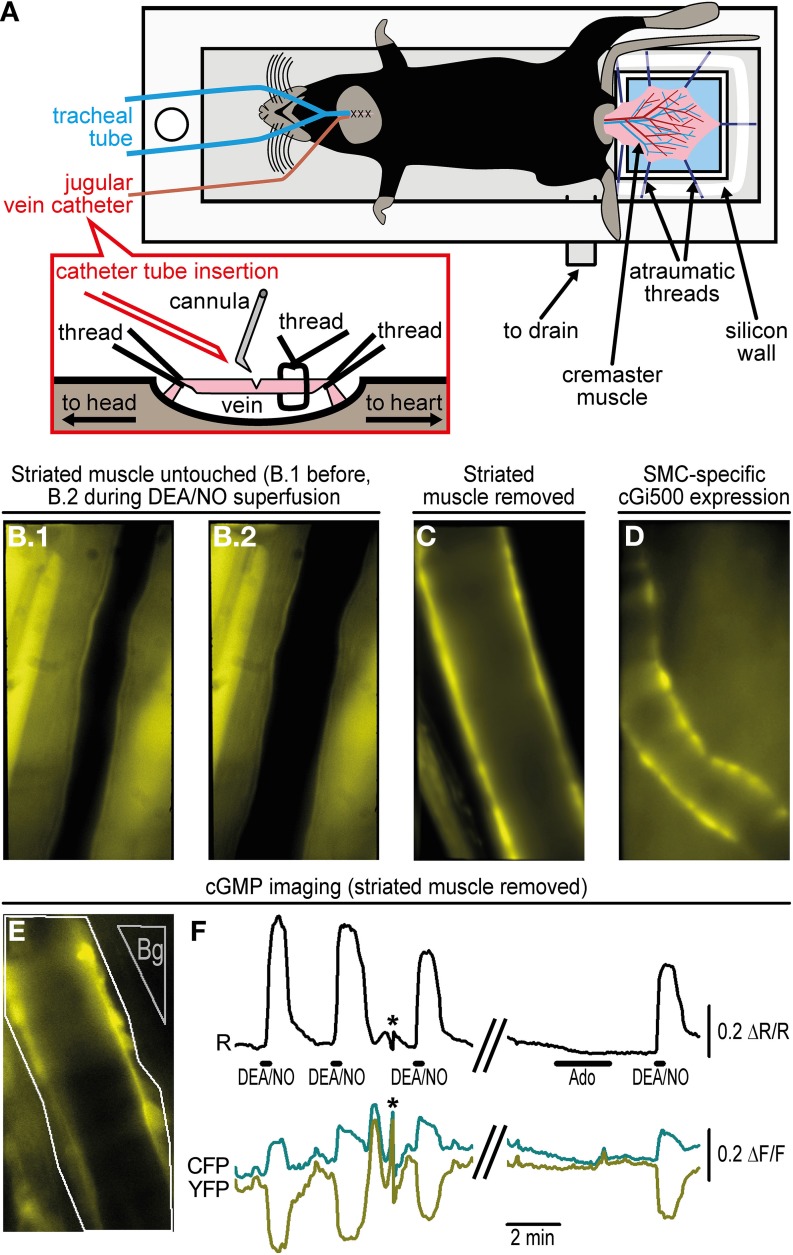
**Imaging of cGMP within the cremaster microcirculation. (A)** A custom-made acrylic glass stage holds the anesthetized mouse in supine position on a heating pad (not shown). The cremaster muscle has been surgically exteriorized and fixed with atraumatic threads on top of a coverslip (light blue). Threads are pinned through a silicone wall that surrounds the coverslip as a semicircle. The cremaster is superfused with pre-warmed (34°C) salt solution delivered along the objective lens (not shown) approaching the cremaster from top. The solution is drained through the outlet at the bottom of the stage. For mechanical ventilation, a tube has been inserted into the trachea. A detailed depiction of jugular vein cannulation is shown in the inset. **(B)** Arteriole within the intact cremaster muscle of a R26-CAG-cGi500(L1) mouse. B.1 shows the vessel before and B.2 during superfusion of 10 μM DEA/NO that causes vasodilation. Estimation of cGMP levels was not possible under this condition, because strong fluorescence from striated muscle prevented FRET measurements within the vessel wall. **(C)** After removal of striated muscle, walls of cremaster arterioles of a R26-CAG-cGi500(L1) mouse are clearly visible, but they lost their basal tone and did not dilate upon superfusion with DEA/NO or other vasoactive drugs. **(D)** In an intact cremaster preparation of a R26-CAG-cGi500(L2); SMA-CreER^T2^ double-transgenic mouse, only the vessel walls show cGi500 expression. The animal was treated for 5 days with 1 mg tamoxifen (i.p.) per day to induce CreER^T2^-mediated activation of the R26-CAG-cGi500(L2) transgene specifically within SMCs. The image was taken 1 week after the last tamoxifen injection. **(E)** Definition of ROIs (arteriole: white outline; background region: gray, “Bg”) for cGMP imaging in the cremaster of a R26-CAG-cGi500(L1) mouse after removal of striated muscle. **(F)** Cyclic GMP imaging of the arteriole shown in **(E)**; the black trace indicates baseline-normalized CFP/YFP ratio changes (ΔR/R) representing intracellular cGMP levels; cyan and yellow traces represent baseline-normalized CFP and YFP fluorescence intensity changes (ΔF/F). Repeated application of 10 μM DEA/NO induced reversible cGMP transients, while 10 μM adenosine (Ado), known to induce relaxation via the cAMP pathway, did not induce detectable ratio changes. Antiparallel changes of CFP and YFP intensities are indicative for true changes of cGi500 FRET efficiency. Asterisks denote artifacts due to focus drift. Acquisition parameters were as follows: excitation: Polychrome V at 420 nm; objective: 40×; acquisition frequency: one image every 0.5 s; camera: Andor iXon 885 with 2 × 2 binning (251 × 501 pixels) and gain set to 25; exposure time: 150 ms. Temporal binning of six frames was done before evaluation performed by subtraction of a background region. Evaluation via image segmentation led to similar results but lower ΔR/R values (not shown). All images **(B–E)** were obtained at 40× original magnification. Panels E and F are reproduced from Thunemann et al. (2013b) and show representative results from experiments with three animals.

1. Anesthesia. Add 0.2 mL midazolam (5 mg/mL, Midazolam-Hameln, Hameln Pharmaceuticals), 0.1 mL dexmedetomidin (0.5 mg/mL Dexdomitor, Lilly Germany), and 0.2 mL fentanyl (0.05 mg/mL Fentanyl-Janssen, Janssen-Cilag) to 2.15 mL Ringer's salt solution (final concentrations: 378 μg/mL midazolam, 19 μg/mL dexmedetomidin, 3.8 μg/mL fentanyl). The animal is anesthetized by intraperitoneal injection of this solution (13 mL/kg body weight) followed by continuous infusion via a jugular vein catheter at a rate of 3 mL/h/kg body weight with a syringe pump (78–9100 W, World Precision Instruments). The infusion rate should be adjusted depending on the sedative effect monitored by provocation tests. If the animal reacts to these tests, increase the infusion rate initially to 4 mL/h/kg body weight, followed by a rate of 3.3 mL/h/kg body weight until the animal no longer reacts.
2. Preparation of surgery. After injection of anesthetics, wait 20 min before shaving the right scrotum, the inguinal region, and the ventral neck region using an electric clipper (GT 420 ISIS, Aeskulap). If the animal still reacts to this manipulation, apply another intraperitoneal injection of anesthetics (5 mL/kg body weight). Place the animal in supine position on a Styrofoam stage that allows temporary fixing of tubes and threads by needles. Maintain and monitor the animal's body temperature by using an electric heating mat and a temperature monitoring system (Telemeter Electronic).
3. Endotracheal tube for mechanical ventilation. Open the neck by a ventral midline incision of ~25 mm length using scissors. The salivary glands covering the trachea are gently pushed aside and the trachea is isolated by blunt preparation and enlaced with two threads. Use these threads to pull the trachea slightly upwards and cut it halfway through with a scalpel to allow the insertion of a tube (15 mm length, I.D. 0.86 mm, O.D. 1.27 mm, polyethylene, Portex, Smiths Medical International). Push the tube 5 mm down the trachea and fix it using the two threads. Ensure that the animal is still breathing and that the knots secure but do not occlude the tube. After the animal has been moved to the imaging setup, connect the tube to the ventilator (Minivent Type 845, Hugo Sachs Elektronik) and start mechanical ventilation (see Section “Imaging”).
4. Jugular vein catheter for intravenous infusion of anesthetics. Locate the jugular vein right to the trachea, clean it carefully from surrounding tissue by blunt preparation, and enlace it using three threads (Figure 2A, inset). Place one thread caudally toward the heart and put it under little tension to obstruct blood flow. The second thread is then positioned cranially, closed, and knotted to obtain a sufficient length of vein between these threads to introduce the catheter tube. Put little tension onto both threads by fixing them temporarily with needles placed into the Styrofoam. Use fine scissors (Fine Science Tools) to open the jugular vein halfway. Use a 27-gauge cannula (0.40 × 20 mm, Sterican, Braun) bend by 90° and attached to a 1-mL syringe to open the vein. Carefully insert a saline-filled polyethylene tube (I.D. 0.26 mm, O.D. 0.61 mm, Portex, Smiths Medical International) beneath the cannula. Release the caudal thread, push the tube about 10 mm toward the heart, and secure the catheter using all three threads. Usually it is possible to verify correct positioning by blood aspiration. Close the wound by three single cutaneous sutures and start continuous infusion of anesthetics at a rate of 3 mL/h/kg body weight as described above.
5. Cremaster preparation. Place the mouse in supine position on a custom-made animal stage that is later used for IVM (Figure 2A). Push the right testicle down into the scrotum. Cut a hole into the scrotum at its peak using scissors, insert one blade, and open the scrotum along its length toward the inguinal region. The cremaster muscle is now visible and requires continuous moistening; start dropping warmed (34°C) salt solution at a rate of 3 mL/min from a reservoir onto the muscle. The salt solution contains (in mmol/L): 118.4 NaCl, 20 NaHCO_3_, 3.8 KCl, 2.5 CaCl_2_, 1.2 KH_2_PO_4_, and 1.2 MgSO_4_; it is gassed with 5% CO_2_ and 95% N_2_ to adjust the pH to 7.4. Mobilize the peak of the muscle pouch and secure it using an atraumatic thread (Serapren 6/0, Serag-Wiessner). Establish a constant fluid flow over the muscle (superfusion) for 10 min to soak and visualize connective tissue. Then, carefully clean the muscle from this connective tissue. Do not cut into muscle tissue and use the initially placed thread to elevate the cremaster to access its bottom side. Now, the muscle pouch is spread over the glass window that is part of the stage. Around this window, a silicon wall holds the threads fixing the edges of the muscle (Figure 2A). Pull the thread attached to the cremaster's peak through the silicon wall and apply some tension to pull the muscle pouch over the window. Use scissors to cut a small hole into the pouch at its peak, insert one blade, and cut it open up to the inguinal region. Avoid cutting through larger vessels that usually lay at the bottom of the pouch. Use 2–3 atraumatic threads at each side to fix the lateral edges of the muscle; apply some tension to flatten the cremaster over the glass window (Figure 2A). Cut the ligament between cremaster and testis to allow repositioning of the testis into the abdominal cavity, which requires cutting one pair of a larger arteriole and vein that enters through this ligament into the testis. Subsequent bleeding will usually cease after a short period. Hemostasis can also be achieved using collagen-based hemostatic sponges.
6. Isolation of arterioles. For particular experiments, arterioles can be freed from surrounding striated muscle (see Result Section “Imaging of cGMP within the Cremaster Microcirculation” and Figure 2C). This procedure requires a surgical microscope with 40-fold magnification (Wild Heerbrugg) and fine preparation tools. Cut into the skeletal fibers that lay on top of the vessels and strip the fibers away using fine forceps. Only isolate small (1–2 mm) arteriole sections for better viability.

#### Imaging

1. Place the animal stage onto the microscope and move the cremaster muscle into the light path. Do not interrupt superfusion of salt solution for longer than 30 s. Attach the ventilator and start mechanical breathing with a frequency of 160 per min and a stroke volume of 225 μL. Check connections of the jugular vein catheter and the level of anesthesia.
2. Move the objective lens to working distance and adjust the rate of superfusion to 8 mL/min. The solution is delivered onto the water immersion objective to form a flowing “drop” between lens and specimen. To provide a physiologic environment, the superfusate is kept at low oxygen tension by gassing with 5% CO_2_/95% N_2_ before and throughout the experiment as well as by using gas-impermeable plastic or glass tubes. Gravity-driven superfusion from a reservoir avoids pulsatile flow, but care has to be taken that the rate of superfusion stays constant throughout the experiment, particularly if drugs are applied through a roller pump (Miniplus3, Gilson). The roller pump delivers drugs into the main superfusion tube just before the fluid reaches the objective. The delivery rate of the roller pump is set to 0.08 mL/min. Thus, drugs delivered by this pump are diluted 100-fold just before they reach the specimen. Vehicle (e.g., water) should be delivered if no drug is applied. Check for continuous removal of the superfusate to avoid leakage into the microscope.
3. Select an appropriate region using bright field and fluorescence illumination. Identify vessel types by diameter, wall structure, and direction of blood flow that diverges in branching arterioles but converges in branching venules.
4. Select illumination time and intensity, as well as camera gain and binning to obtain images with sufficient signal-to-noise ratios, while avoiding excessive photobleaching. Settings depend on the imaging setup as well as the biosensor that is used, and its expression level. Parameters have to be optimized for individual setups and according to experimental demands (e.g., temporal vs. spatial resolution). Representative acquisition parameters for the setup described in Section “Imaging setup” are given in the legend to Figure 2F.
5. Acquire time series under basal conditions to estimate signal-to-noise levels and to observe potential baseline drift or basal activity of the cells under investigation.
6. After a stable baseline is recorded for a sufficient period (e.g., for 30–60 frames), drugs (e.g., the NO-releasing drug DEA/NO) can be applied via superfusion using the roller pump. Exchange vehicle with drug solution to start application and back to vehicle to terminate application. The time delay until the drug reaches the specimen can be estimated by sucking air into the tube of the roller pump. Observe the air bubble moving through the tube and measure the time until its delivery.
7. During the imaging session, check anesthesia and vital parameters from time to time. Based on our experience, IVM can be performed for up to 6 h. At the end of the experiment, apply a lethal dose of pentobarbital (1 g/kg body weight, e.g., 6.25 mL/kg body weight of a 160 mg/mL pentobarbital solution, Narcoren, Merial) through the jugular vein catheter.

### Multiphoton cGMP imaging in subcutaneous vessels using the dorsal skinfold chamber

#### Imaging setup

Details on this IVM setup have been described previously (Brown et al., 2001).

- Microscope: upright BX51WI (Olympus) with Fluoview FV300 scanning system and 20×/0.95 N.A. water immersion lens. The system is placed on a vibration-free table (Melles Griot) within a light-tight enclosure.
- Light source: MaiTai Ti:Sapphire laser (Spectra-Physics) providing 100 fs pulses at 80 MHz repetition rate and 850 nm wavelength with a power of 36 mW at the sample surface.
- Filters: 750 nm short pass dichroic mirror (Chroma). FRET filter set: 505 nm long pass dichroic mirror (Chroma), 483/32 nm and 542/27 nm emission filters (Semrock).
- Detection: HC125-02 photomultiplier tubes (Hamamatsu Photonics).
- Software: FluoView (Olympus).

#### Surgery

All surgical procedures have to be approved by the Institutional Animal Care and Use Committee. The procedure follows the protocol for implantation of dorsal skinfold chambers (DSCs) in mice as described in Leunig et al. (1992).

**Figure 3:**
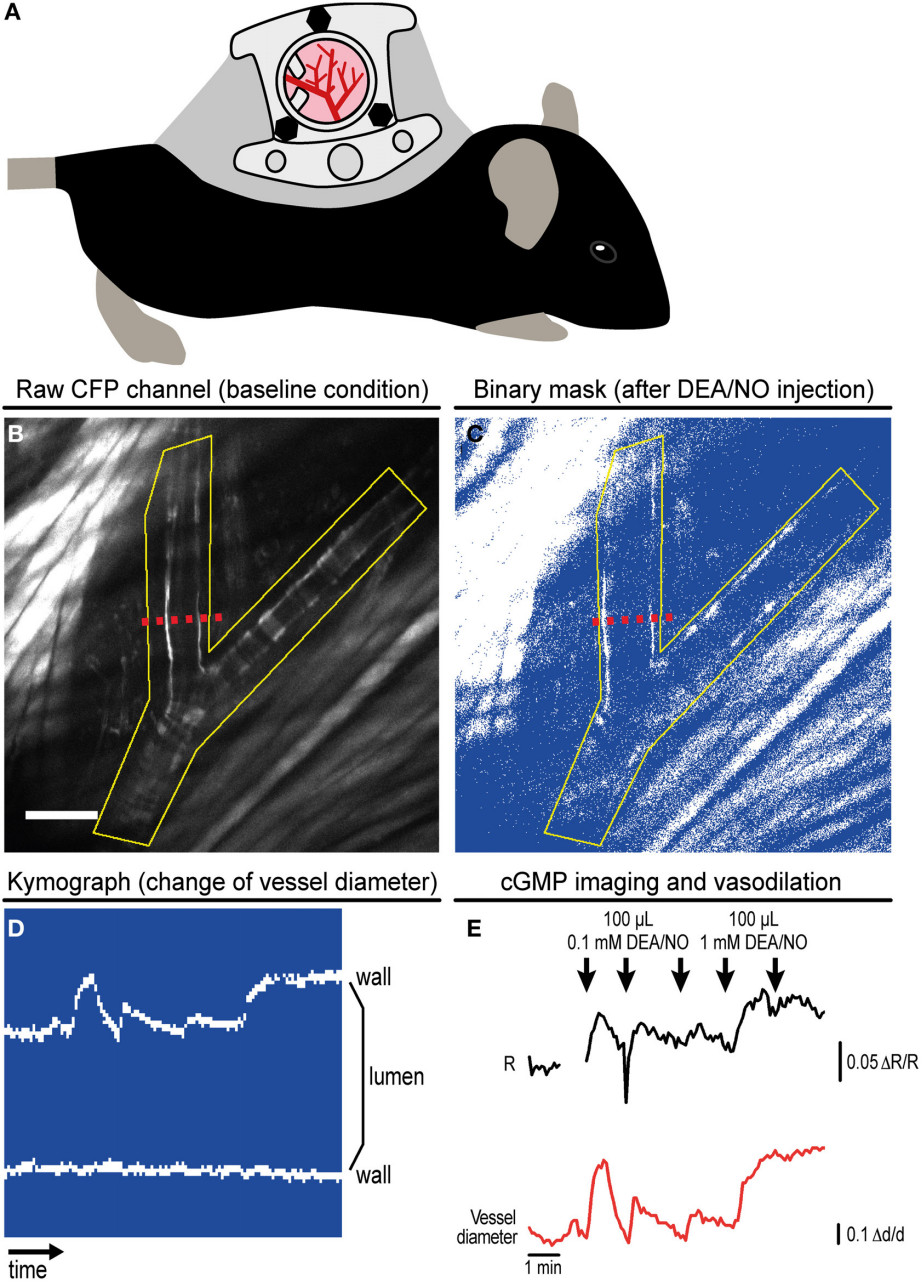
**Imaging of cGMP in subcutaneous vessels using the dorsal skinfold chamber (DSC). (A)** Schematic drawing of a mouse with implanted DSC. **(B)** Field of view observed with MP microscopy through the DSC used for cGMP imaging as shown in **(C–E)**. **(B)** shows the tissue before DEA/NO injection. Scale bar: 100 μm. **(C)** Image from the dynamic binary mask showing the tissue shortly after DEA/NO injection. **(D)** Kymograph showing movements of the vessel wall at the cross-section indicated by dashed red lines in **(B,C)** (time increases from left to right). Vasodilation was solely caused by movement of the upper vessel wall (left wall in **B,C**). **(E)** Cyclic GMP imaging of the (yellow) ROI outlined in **(B,C)**. The black trace shows changes of the baseline-normalized CFP/YFP ratio (ΔR/R) upon three intravenous injections of 0.1 mM DEA/NO followed by two injections of 1 mM DEA/NO. Few time points were omitted due to disturbance of image acquisition by ambient light. The relative vessel diameter change (Δd/d, red trace) was determined from the kymograph shown in **(D)**. Acquisition parameters were as follows: excitation: 850 nm with 36 mW at the tissue surface; objective: 20×; acquisition frequency: one image every 5 s; image size: 512 × 512 pixels (707.11 × 707.11 μm). **(B,E)** are reproduced from Thunemann et al. (2013b). Representative results from five experimental sessions with three animals are shown.

1. Anesthetize mice by intraperitoneal injection of 90 mg/kg body weight ketamine and 9 mg/kg body weight xylazine. Wait ~15 min until the animal does not react to provocation tests anymore. During the surgical procedure, maintain the animal's core body temperature at 36–37°C using a heating pad. Apply a lubricant to prevent corneal dehydration.
2. Shave and depilate the entire back of the animal prior to chamber implantation.
3. Gently stretch the double layer of the skin from the back of the mouse and implant two symmetrical titanium frames (weight 3.2 g; produced at the workshop of the Department of Radiation Oncology, MGH) making mirror images of each other to sandwich the skin. Place sutures in the top part of the skin through the holes at the top edge of the titanium frames to hold the frames in place.
4. Remove one layer of the skin in a circular area of 15 mm in diameter. To stop bleeding, apply gentle pressure using a cotton swab. Cover the remaining layer consisting of epidermis, subcutaneous tissue, and striated muscle with a glass coverslip incorporated into a depression in one of the frames. Hold the coverslip in place with a retaining ring (Figure 3A).
5. Allow the animal to recover for at least 48 h. For analgesia, administer 0.05–0.1 mg/kg body weight buprenorphine every 12 h subcutaneously for 3 days, and thereafter as needed. Animals loose about 15% of their body weight within the first 48 h after DSC implantation and further weight loss or other signs of distress are usually not observed (Leunig et al., 1992). If there is any sign of inflammation, redness, fluid accumulation, edema, etc. in the DSC, animals are further observed for recovery or excluded from the study.

#### Imaging

IVM has to be performed with approval by the Institutional Animal Care and Use Committee.

1. Anesthetize a mouse with DSC by subcutaneous or intraperitoneal injection of ketamine/xylazine (90/9 mg/kg body weight). Alternatively, gas anesthesia such as 1–1.5% isoflurane in medical air can be used. Properly maintained gas anesthesia is more stable and suitable for long and/or physiological imaging studies.
2. Place the anesthetized animal on a heated microscope stage. Apply a lubricant to prevent corneal dehydration. Clamp the chamber to a custom-built metal frame on the microscope stage. For drug application, place an intravenous catheter connected to a 1-mL syringe filled with 0.9% NaCl into the tail vein. The catheter consists of a 30-gauge needle connected to polyethylene tubing (PE-10, BD Medical, I.D. 0.28 mm, O.D. 0.61 mm) of appropriate length (see below). Fix the catheter needle with a small drop of superglue to the tail and the tubing with adhesive tape to the microscope stage.
3. Place the animal-bearing stage on the imaging setup. The syringe connected to the catheter must later be accessible without disturbing image acquisition. Place a drop of water on the coverslip of the DSC and move the objective into working distance. Select an appropriate region under bright field illumination. Check the animal's vital parameters and close the enclosure. Examine the field of view under fluorescence illumination and fine-adjust x/y-position and the z focus.
4. Prepare image acquisition: adjust illumination intensity, scan speed, zoom, and photomultiplier voltage to obtain images with sufficient signal-to-noise ratios. Image acquisition may not cause excessive bleaching of the biosensor. Settings have to be optimized according to the IVM setup, the respective biosensor and its expression level, as well as experimental demands (e.g., temporal vs. spatial resolution). Representative acquisition parameters for the setup described in Section “Imaging setup” are given in the legend to Figure 3E.
5. Acquire time series under basal conditions to estimate signal-to-noise levels and to observe potential baseline drifts or basal activity of the cells under investigation.
6. After a stable baseline is recorded for a sufficient period (e.g., for 20–30 frames), substances are applied through the tail vein catheter. Apply vehicle (saline or PBS) and then test compounds (e.g., the NO-releasing drug DEA/NO); consider the catheter's death volume.
7. Ketamine/xylazine anesthesia typically lasts for 30–40 min; check regularly for vital functions and state of consciousness during the imaging session. If necessary, inject ketamine again; use half of the initial dose. The animal should not be anesthetized for more than 1 h. After imaging, carefully remove the catheter from the tail and allow the animal to recover in its home cage. We recommend performing imaging experiments not more frequently than every other day.

### Image segmentation with a binary mask and data evaluation

Image processing is performed using the open source software ImageJ or ImageJ-derived software (Schneider et al., 2012). In establishing this protocol, the data were evaluated manually, although ImageJ and other software packages (e.g., Metamorph, IDL, Matlab) have built-in functions and routines for automation. Handling of time-lapse image series may require large amounts of free working memory; therefore, data evaluation should be performed on computers equipped with ≥8 GB RAM.

**Figure 4:**
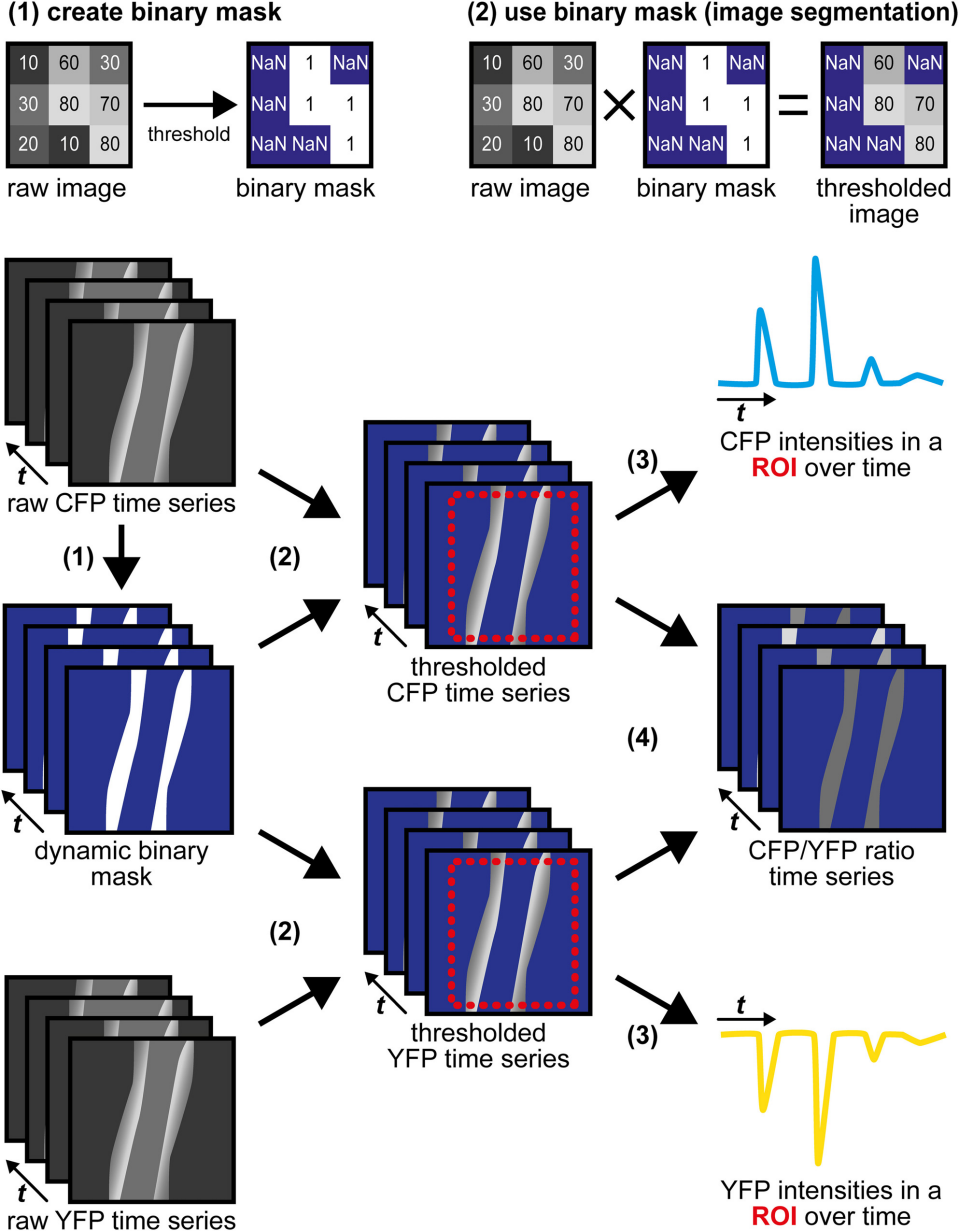
**Image segmentation approach to evaluate time-lapse series of moving structures**. (1) A threshold is applied to the raw CFP time series to generate a dynamic binary mask. The raw YFP series can also be used, but only one mask (from CFP or from YFP) is required. Numbers within the raw images represent virtual pixel intensities; in this example, the threshold was set to an intensity of 50. Now, target structures with intensities above the threshold receive the value “1” (white), while dim/non-fluorescent structures below the threshold receive the value “NaN” for “not a number” (blue), that does not equal “0.” (2) Image segmentation is performed by multiplication of the same dynamic binary mask with the original raw CFP and YFP time series. Within segmented time series, pixels representing (bright) target structures retain their original intensities, while pixels representing dim/non-fluorescent structures are now set to non-numerical values (“NaN,” blue). (3) CFP and YFP intensity changes are extracted from segmented time series in a region of interest (ROI, red) that contains the moving vessel wall throughout the whole time series. “NaN” pixels do not contribute to the ROI's mean intensities. (4) Segmented CFP and YFP time series are used to calculate pixel-by-pixel CFP/YFP ratio series. As background is set to “NaN,” ratio series do not suffer from artifacts occurring when noisy images are divided by each other. The dynamic binary mask can also be used to estimate changes in vessel diameter (see Figure 3D).

1. Image preprocessing. Save CFP and YFP time series as files readable by ImageJ (http://imagej.nih.gov/ij/) or ImageJ-derived distributions like Fiji (http://fiji.sc/Fiji). Open the time series and check for correct alignment of CFP and YFP channels. If necessary, apply corrections like manual or automatic alignment procedures (see, e.g., Kardash et al., 2011; Pitkeathly et al., 2012). Signal-to-noise ratios can be improved by spatial or temporal binning (method: “sum” or “average”) that on the other hand will lead to loss of spatial or temporal resolution. Thereafter, intensities from a background region can be subtracted and baseline normalization can be performed as described previously (Thunemann et al., 2013a). To account for tissue motion during image acquisition, we suggest performing image segmentation.
2. Image segmentation with a dynamic binary mask. This routine derived from a strategy originally described by Zhang et al. (2010) is performed to follow CFP/YFP ratio changes in moving structures, such as the vessel wall during vasodilation. Subsequent steps and Figure 4 describe how the dynamic binary mask is generated und used:
  - Apply the “threshold” function to the raw CFP or the raw YFP time series; select the lower threshold so that the structure of interest is above the threshold throughout the whole time series (perform “stack histogram function”). If CFP and YFP time series differ in quality (e.g., in signal-to-background ratios), use the series of higher quality, but make sure that decreased fluorescence caused by FRET efficiency changes do not cause the structure of interest to fall below the threshold.
  - The final binary mask is generated by dividing the thresholded time series by itself using the “Image Calculator” function with the “32-bit (float) result” option enabled. The resulting dynamic binary mask has only values of “1” (pixels *above* threshold) or “NaN” for “not a number” (pixels *below* threshold).
  - Apply this mask to both time series using the “Multiply” operation within the “Image Calculator” function. In the resulting segmented CFP and YFP time series, bright structures above the threshold possess intensity values as in the original time series, while dim/non-fluorescent structures below the threshold have “NaN” values (Figure 4).
  - Note that background fluorescence (set to “NaN”) is not subtracted from pixels above the threshold (see also discussion in Section “Multiphoton cGMP Imaging of Subcutaneous Vessels Using the Dorsal Skinfold Chamber”).
3. ROI definition and extraction of CFP and YFP intensities. Draw regions defining the target structure during the whole experiment considering shape changes and movements. As “NaN” pixels do not contribute to the region's average intensities, ROIs may contain large proportions of background fluorescence. Add regions to the “ROI Manager” and perform the “Multi Measure” command twice to extract mean intensities from both channels. Transfer values to Excel (Microsoft) or Origin (OriginLab) for further evaluation as described (Thunemann et al., 2013a).
4. Generation of time-lapse ratio images. Pixel-by-pixel CFP/YFP ratio series representing cGMP levels are calculated by dividing thresholded time series by each other using the “Image Calculator” function.
5. Estimation of vessel diameter. To estimate changes in vessel diameter, draw a line orthogonal to the direction of blood flow into the dynamic binary mask. The line has to cover both vessel walls throughout the experiment. Generate a kymograph using the “Reslice” command within the “Stacks” menu with the “Rotate by 90 degrees” option enabled. The resulting image shows the time series at the cross-section of the vessel wall with time increasing from left to right (Figure 3D) and can be used for manual or semi-automatic vessel diameter estimation. Kymographs derived from CFP/YFP ratio series show changes in cGMP levels and vessel diameter within the same image.

## Results and discussion

### Selection of appropriate biosensors and generation of biosensor-expressing mice

Two designs of genetically encoded biosensors are commonly used. One class includes monomolecular FRET-based biosensors like cGi-type cGMP sensors that contain donor and acceptor fluorophore in a single polypeptide chain. Other biosensors, such as FlincG-type cGMP sensors (Nausch et al., 2008; Bhargava et al., 2013) or Ca^2+^ sensors of the GCaMP family (Tian et al., 2009; Chen et al., 2013), contain circularly permutated green fluorescent protein (cpEGFP). Monomolecular FRET-based biosensors can be measured by simultaneous acquisition of donor and acceptor emission upon donor excitation. In camera-based systems, beam splitters should be used to direct donor and acceptor fluorescence to two separate cameras or onto the halves of a single camera chip. In scanning microscopes, emitted light is detected by two separate photomultiplier tubes. Emission of cpEGFP-based biosensors can be measured at a single wavelength. Potential advantages and disadvantages of FRET- and cpEGFP-based biosensors are discussed in more detail in Newman et al. (2011), Kaestner et al. (2014). Ideally, any change in biosensor fluorescence should originate from ligand binding, but not from other processes that affect fluorescence intensity recordings, such as motion of the target structure. In this regard, ratiometric FRET-based biosensors are advantageous over cpEGFP-based biosensors, because not a single fluorescence intensity (e.g., EGFP) but the ratio between two simultaneously recorded fluorescence intensities (e.g., CFP/YFP) is taken to estimate ligand binding to the sensor. Artificial intensity changes will not affect quantification as long as both donor and acceptor fluorescence are affected to the same extent. This should be the case, for instance, upon movement of the imaged tissue due to vasodilation, heartbeat, blood flow, or breathing.

Biosensor-expressing transgenic mice are important tools for IVM. Expression of the biosensor must be appropriate for its detection with sufficient signal-to-noise ratios. Initially, we used random transgenesis to generate cGi500-expressing mice, which is a relatively simple method in terms of transgene assembly and mouse generation. However, it often requires screening of many founder lines to identify lines with stable and sufficient transgene expression. Using an appropriate promoter is critical for successful generation of such a transgenic mouse line. Unfortunately, random integration of the transgene into the genome often leads to unpredictable and unwanted expression patterns. We used the 445-bp promoter fragment of the Transgelin/SM22 gene (Li et al., 1996) to generate SM22-cGi500 mice with SMC-specific cGi500 expression (Figure 1C upper). Among 11 founder lines, one mouse line showed stable SMC-specific cGi500 expression (data not shown). The expression level of cGi500 in SMCs derived from aorta, colon, and bladder is heterogeneous with ~25% of the cells showing relatively strong cGi500 expression (Thunemann et al., 2013b). However, SM22-cGi500 mice do not express detectable cGi500 levels in cremaster arterioles (Thunemann et al., unpublished data), so that these mice cannot be used for cGMP imaging in the cremaster microcirculation.

**Figure 5:**
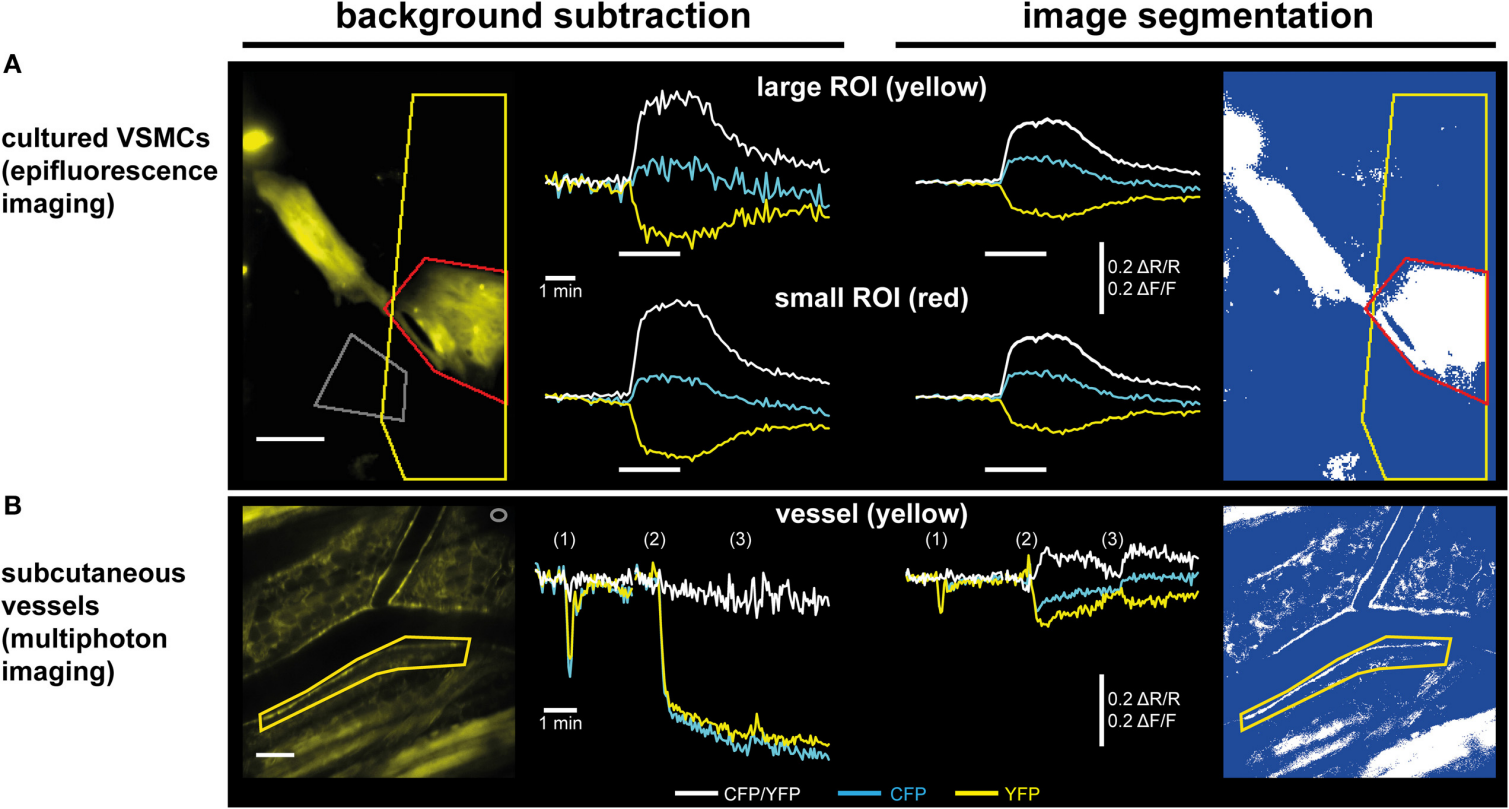
**Subtraction of a background region vs. image segmentation for evaluation of ratiometric FRET/cGMP imaging data**. Traces show baseline-normalized CFP/YFP ratio changes (ΔR/R; white) or baseline-normalized CFP and YFP intensity changes (ΔF/F; cyan and yellow, respectively). **(A)** Evaluation of epifluorescence recordings of cGi500-expressing cultured vascular smooth muscle cells (VSMCs) isolated from R26-CAG-cGi500(L1) mice. White bars indicate superfusion with 100 nM DEA/NO. Upon subtraction of a background region (left, gray ROI), selection of a large ROI (yellow) containing a high proportion of dim/non-fluorescent background increases noise (upper left) as compared to a small ROI (red) containing mainly the fluorescent cell (lower left). Applying image segmentation (right, see Figure 4 for further details) with the same ROIs results in lower ΔF/F and ΔR/R changes. The noise levels are not affected by the proportion of dim/non-fluorescent background within a ROI and thus, results are the same for small and large ROI. Scale bar, 50 μm. **(B)** Evaluation of *in vivo* FRET/cGMP recordings obtained from subcutaneous vessels via MP microscopy through a DSC; numbers in brackets indicate bolus drug applications: (1): 100 μL 0.9% NaCl; (2), and (3): 100 μL 1 mM DEA/NO. The image was taken right before the first DEA/NO injection. Subtraction of a background region (left, gray ROI in top right corner) is not suitable for evaluation, because DEA/NO-induced vasodilation causes massive drops in fluorescence intensities due to an increased contribution of dim/non-fluorescent vessel lumen to the ROI defining the vessel wall (yellow). This large drop obscures comparably small alterations in CFP and YFP fluorescence caused by cGMP-induced changes in FRET efficiency. When image segmentation is used (right), dim/non-fluorescent structures do not contribute to mean ROI intensities, leading to much smaller drops in CFP and YFP fluorescence, and cGMP-induced ΔR/R changes are revealed. Scale bar: 100 μm. **(B)** is modified from Thunemann et al. (2013b).

To generate mice with strong and homogeneous cGi500 expression, we used targeted mutagenesis to integrate a Cre-dependent cGi500 expression cassette into the R26 gene locus (Figure 1C middle). This gene locus is known for transcriptional accessibility in most if not all cell types throughout pre- and postnatal development (Soriano, 1999). Although technically more challenging, generation of R26 knock-in mice became a popular approach to express fluorescent proteins including biosensors (Zariwala et al., 2012; Abe et al., 2013; Batti et al., 2013). To increase biosensor expression above the relatively low levels that are usually achieved with the endogenous R26 promoter in adult mice, the strong chicken actin/β-globin (CAG) promoter (Niwa et al., 1991) can be integrated into the R26 knock-in transgene. Activation of sensor expression is achieved by breeding mice carrying the R26 knock-in transgene to cell type-specific Cre-transgenic mouse lines. This strategy should allow one to direct high and homogeneous levels of biosensor expression to almost any cell type of interest. We have generated R26 knock-in mice carrying the Cre-dependent R26-CAG-cGi500(L2) transgene (Thunemann et al., 2013b). We observed strong and tissue-specific cGi500 expression in mice generated by breeding R26-CAG-cGi500(L2) mice with tissue-specific Cre mice (Figure 2D, and Wen, Thunemann, Feil, unpublished results). This finding is in line with numerous reports on other Cre-inducible R26 knock-in transgenes (Casola, 2010). Furthermore, we transfected embryonic stem cells carrying the R26-CAG-cGi500(L2) transgene with a Cre expression plasmid to generate the permanently activated R26-CAG-cGi500(L1) transgene (Figure 1C lower). Indeed, mice established from R26-CAG-cGi500(L1) embryonic stem cells showed strong cGi500 expression in virtually all tissues and cell types examined (Thunemann et al., 2013b). Importantly, use of the CAG promoter resulted in high cGi500 expression levels and sufficient fluorescence intensities for *in vivo* imaging experiments (Figures 2, 3, 5). We conclude that the wide use of the Cre/lox system and the availability of hundreds of Cre mouse lines combined with the positive experience using the R26 locus as knock-in target, the generation of biosensor-expressing mice via Cre/lox-activatable R26 knock-in transgenes is an attractive alternative to random transgenesis.

When working with biosensor-expressing cells, tissues, and mice, it is important to consider potential side effects of sensor expression. A biocompatible sensor should not interfere with normal physiology. The cGi500 sensor contains the cGMP-binding sites of cGK type I, but not its catalytic kinase domain nor its N-terminal region that interacts with various proteins (Figure 1B). Although we cannot formally exclude that cGi500 acts as an intracellular cGMP buffer, we did not observe any adverse effects of its expression on the health of cGi500-transgenic mice nor on the functions of cells isolated from these mice (Thunemann et al., 2013b). In this context, it is interesting to note that mice expressing a FRET-based Ca^2+^ sensor in a ubiquitous manner show only relatively mild phenotypes (Direnberger et al., 2012). However, when mice express a biosensor with enzymatic activity, for instance, myosin light chain kinase activity in MLCK Ca^2+^ indicator mice (Isotani et al., 2004), it might be important to exclude interference of the sensor with normal physiology by reducing its expression level as much as possible.

### Imaging of cGMP within the cremaster microcirculation

The mouse cremaster is a relatively transparent skeletal muscle (~200 μm thick) that forms a pouch around the testis and contains resistance-type vessels. The first *in vivo* study of vascular responses within the cremaster was published in 1964 (Grant, 1964). Most current protocols for cremaster IVM, including those presented herein, follow the procedure described by Baez (1973), which includes the opening of the cremaster muscle, its detachment from the testicle, and the spreading of the cremaster to a flat sheet (Figure 2A). A number of studies used biosensors to visualize Ca^2+^ in endothelium and SMCs of cremaster arterioles. However, the functional role of cGMP in the cremaster microcirculation has so far been studied only indirectly, for instance, by analyzing vasodilation or vascular permeability in mouse lines with mutations of components of the cGMP signaling pathway (Koeppen et al., 2004; Chen et al., 2012).

Using the cGi500 sensor, we were able to visualize changes in cGMP concentrations in cremaster arterioles *in vivo* (Thunemann et al., 2013b). Experiments were performed with the R26-CAG-cGi500(L1) mouse line showing ubiquitous cGi500 expression. Accordingly, we observed cGi500 expression in the vessel wall and in the striated muscle of the cremaster itself, in which vessels are embedded, as well as in circulating blood cells (Figures 2B,C,E, and data not shown). Application of the NO-releasing drugs sodium nitroprusside and DEA/NO, as well as adenosine and acetylcholine induced dilation of cremaster arterioles (Figure 2B, and data not shown). However, attempts to detect DEA/NO-induced cGMP signals in arterioles of the intact cremaster preparation (as shown in Figure 2B) failed due to strong cGi500 fluorescence in striated muscle (data not shown). After surgical removal of the skeletal muscle from arterioles (Figures 2C,E), robust CFP/YFP ratio changes indicating reversible cGMP elevations could be observed in the vessel wall upon superfusion of DEA/NO, while adenosine did not cause a detectable cGMP elevation (Figure 2F). However, because vessels that were isolated from surrounding tissue apparently lost their basal tone and did not dilate upon drug superfusion, this experimental setup did not allow us to correlate cGMP levels with the extent of vasodilation. Moreover, because of ubiquitous cGi500 expression in R26-CAG-cGi500(L1) mice, it is difficult to allocate cGMP signals to specific vascular cell types. Thus, this model has two limitations related to ubiquitous sensor expression. Firstly, FRET recordings are disturbed by fluorescence from striated muscle and, secondly, it is difficult to pinpoint cGMP signals to specific vascular cell types. Both problems could be avoided by cell type-specific cGi500 expression within distinct cell types of the cremaster microvasculature. Therefore, the Cre-responsive R26-CAG-cGi500(L2) mouse line is currently bred with endothelium- or SMC-specific Cre lines (Figure 2D, and data not shown). Furthermore, by using other Cre-transgenic lines, it should be possible to visualize cGMP signals in various other cell types, for instance, in leukocytes during inflammatory processes or in platelets during adhesion to the vessel wall and thrombus formation.

Advantages of the open cremaster model for IVM are its outstanding optical quality and relatively minor motion artifacts. The good accessibility of arterioles within the exteriorized cremaster allows direct drug delivery via superfusion or locally via glass pipettes. Applying drugs only to the tissue of interest and not systemically through intravenous infusion can be an advantage. Continuous superfusion almost instantaneously removes drugs, allowing for studies with many successive drug applications, for example, to generate concentration-response curves or to compare effects of different drugs. On the other hand, cremaster IVM requires supported breathing, continuous delivery of anesthetics, temperature maintenance with external heat sources, and constant superfusion with gassed salt solution. The time required to prepare an animal for imaging is around 1 h, and the time for experiments is limited to about 6 h per animal.

### Multiphoton cGMP imaging of subcutaneous vessels using the dorsal skinfold chamber

The DSC model (Figure 3A) has been established to study tumors implanted below the chamber window. In addition, this method allows imaging of the tumor environment, for instance, to monitor changes in vascularization during tumor growth (Brown et al., 2001). IVM studies with DSCs were initially performed with bright field, epifluorescence, or confocal microscopy, but nowadays, MP microscopy is primarily used. MP excitation has several advantages over single photon excitation: out-of-focus fluorescence above or below the focal plane is avoided, excitation with pulsed infrared lasers allows for larger penetration depths (up to ~0.5 mm tissue), and the infrared light causes comparatively mild damage to fluorescent molecules and surrounding tissue (Helmchen and Denk, 2005).

We implanted DSCs into R26-CAG-cGi500(L1) mice and used them for MP-FRET/cGMP imaging (Thunemann et al., 2013b). Subcutaneous vessels were readily visible and, in contrast to the cremaster model, we were able to identify regions where surrounding tissue did not interfere with the detection of FRET/cGMP signals from vessel walls (Figures 3B,E). Mice received vasoactive drugs as bolus injections via a tail vein catheter. In our experiments, 3–5 drug applications were performed over 15–30 min. In studies with cultured cells and cremaster arterioles, the initial steps in image evaluation are ROI definition and subtraction of a dim/non-fluorescent background region (see Figure 2E). When we subtracted intensities from a background region, vasodilation strongly confounded the analysis of CFP/YFP ratio signals within the vessel wall (Figure 5B left). Vasodilation led to an increase in the proportion of dim/non-fluorescent vascular lumen within the ROI that resulted in strong and parallel reductions of CFP and YFP fluorescence. These drops were much stronger than any intensity changes caused by cGMP binding to cGi500 (Figure 5B left). To overcome this problem, we performed image segmentation with a dynamic binary mask (Figure 4). Here, instead of subtracting a background region, structures with a brightness below a certain threshold are set to non-numeric values (“NaN”). These structures actually can be fluorescent; however, their brightness is lower compared to the target structure, i.e., the vessel wall. To compare subtraction of a background region and image segmentation, we applied both methods to cGi500 recordings in cultured cells. Subtraction of a background region results in greater changes of the baseline-normalized CFP/YFP ratio (ΔR/R) as compared to ratio changes obtained through image segmentation (Figure 5A, compare left and right traces), most likely as a direct result of different background treatment. However, when the dynamic binary mask is used, varying proportions of dim/non-fluorescent background within the ROI do not affect noise levels (Figure 5A, compare right traces from small and large ROI), which can be an advantage over the background subtraction method (Figure 5A, compare left traces from small and large ROI). Consequently, ROIs can now be defined to contain large and/or variable proportions of dim/non-fluorescent background without affecting signal quantification. Using this approach with DSC recordings markedly reduced the confounding drop of CFP and YFP fluorescence upon vasodilation that was observed when a background region was subtracted (Figure 5B). This alternative evaluation strategy eventually allowed us to detect cGMP transients in the vascular wall during vasodilation *in vivo*. Control injection of saline caused a transient vasodilation, most likely due to an increase in blood volume (data not shown) that was not associated with CFP/YFP ratio changes [Figure 5B, right, application (1)]. Injection of DEA/NO caused vasodilation (Figure 3E) that was associated with a clear ΔR/R increase indicating an increase of cGMP levels [Figures 3E and 5B, right, applications (2) and (3)]. Relative vessel diameters were extracted from dynamic binary masks via kymographs (Figure 3D). The extent of NO-induced cGMP elevations measured with the cGi500 sensor correlated well with the extent of vasodilation (Figure 3E). Thus, data evaluation with the image segmentation method made it possible to observe cGMP transients in dilating vessels (Figures 3B–E).

The highest CFP/YFP ratio changes observed in the DSC model were relatively moderate (~10%) compared to changes recorded in the cremaster microvasculature (~50%). Although we cannot exclude biological reasons, this discrepancy is likely caused by different ways of sensor excitation (single photon vs. MP) and data evaluation (treatment of background fluorescence). While image segmentation of DSC recordings was performed without background subtraction, non-specific fluorescence from a background region was subtracted in cremaster recordings. As Figure 5 clearly shows, the evaluation strategy strongly affects ΔR/R values. Therefore, quantitative comparisons are only feasible between experiments evaluated with the same strategy. Note that omitting background subtraction during image segmentation may introduce variability if the background varies considerably between experiments. We are working on further refinements of our evaluation approach and expect substantial improvements with the use of mice expressing cGi500 in a cell type-specific manner (see Figure 2D). Another difference was that single-photon excitation was used for cremaster imaging and MP excitation in the DSC model. For the latter, we tested excitation wavelengths between 820 and 880 nm and we have chosen 850 nm for best imaging results (data not shown). Due to overlapping excitation spectra of CFP and YFP and the non-linearity of MP excitation, MP lasers likely cause direct excitation of the acceptor fluorophore, YFP (Zipfel et al., 2003; Svoboda and Yasuda, 2006). We assume that direct YFP excitation as well as bleed-through of CFP emission into the YFP channel were, at least in part, responsible for the relatively low ΔR/R values observed during MP imaging in the DSC model. In order to apply correction algorithms for tissue-intrinsic background fluorescence or spectral bleed-through, or to perform spectral unmixing, samples containing either no, or only the donor or the acceptor fluorophore need to be recorded (Van Rheenen et al., 2004; Thaler et al., 2005; Thaler and Vogel, 2006; Zhang et al., 2010; Zeug et al., 2012; Mauban et al., 2014). In addition, alternative methods to estimate FRET efficiencies such as fluorescence lifetime imaging microscopy (FLIM) are available (Yasuda et al., 2006; Levitt et al., 2009; Fruhwirth et al., 2011). However, recording appropriate donor- or acceptor-containing specimens, or using FLIM techniques with non-optimized FRET pairs can be challenging under *in vivo* conditions (Niesner and Hauser, 2011; Geiger et al., 2012; Kamioka et al., 2012).

In the DSC model, animals can be analyzed repeatedly for up to 5 weeks, which is an advantage over the cremaster model. The DSC model allows longitudinal studies in individual animals, for example, to follow disease progression. Thereby, animal numbers can be reduced, while statistical power is maintained. Another advantage over the cremaster model is that tissues within the DSC are not acutely touched when imaging is performed. Under the conditions described here, test drugs are delivered intravenously. Due to volume restrictions, this limits the number of drug applications during an imaging session to about three to five. Depending on the experimental question, systemic drug delivery might complicate data interpretation. If so, the DSC model can also be used for direct topical application of test compounds. To this end, the coverslip is carefully removed to visualize vessels and other structures while applying solutions that contain test compounds (Fukumura et al., 1997). A number of protocols exist to implant windows at different sites of the body (Jain et al., 2010; Ritsma et al., 2012). This flexibility allows studying cGMP and other signaling molecules in diverse vascular beds, such as cerebral vessels and the vasculature of abdominal or thoracic organs. For these experiments, the setup has to reach sufficient scanning speeds to resolve the kinetics of the biochemical/physiological processes under investigation. Acquisition speed is especially important when moving cells (e.g., in the blood) or structures (e.g., the beating heart) are analyzed. Here, camera-based systems have an advantage as they typically allow faster acquisition rates (Stephens and Allan, 2003). For some IVM applications, spinning disk systems might be a suitable alternative to MP systems (Jenne et al., 2011), particularly when combined with MP excitation, thereby achieving large penetration depths and fast acquisition rates (Shimozawa et al., 2013). Alternatively, resonant galvanometer mirror scanning enables MP microscopy at high speeds to monitor fast biological processes (Kirkpatrick et al., 2012).

## Conclusion and outlook

Correlative IVM of biochemical processes and physiological responses is a powerful technique to study cardiovascular biology *in vivo*. Although technically challenging, experiments performed in living animals are essential to analyze the functional relevance of proteins and signaling molecules in health and disease. We have generated and characterized transgenic mice expressing the FRET-based cGMP biosensor cGi500 in the vasculature. Indeed, intravital FRET imaging combined with an image-processing routine that accounts for tissue motion allowed us to monitor cGMP signals and associated vasodilation simultaneously in living mice. These and similar mouse models should be useful to investigate the crosstalk between vascular cGMP, cAMP, and Ca^2+^ signaling. For these and other studies, it will be important to proof the selectivity of the cGi500 sensor for detection of cGMP over cAMP *in vivo*, for example, by using cAMP-increasing agents and pharmacological/genetic inhibition of NO-induced cGMP synthesis. In the future, cGi500-expressing mice can be used to visualize cGMP selectively in defined cell types of the vessel wall, such as endothelial cells or SMCs, or to analyze cGMP signaling in mouse models of cardiovascular disorders. Another step to take is the precise quantification of cGMP concentrations within the vasculature under basal and stimulated conditions *in vivo*, as it has recently been described for Ca^2+^ (Mauban et al., 2014). The combination of genetic mouse models, optical biosensors, and state-of-the-art IVM methods should soon allow us to “watch” biochemistry, (patho-)physiology, and pharmacotherapy in the context of a living mammalian organism.

### Conflict of interest statement

The authors declare that the research was conducted in the absence of any commercial or financial relationships that could be construed as a potential conflict of interest.
